# Epithelial–Mesenchymal Transition Participates in the Formation of Vestibular Flat Epithelium

**DOI:** 10.3389/fnmol.2021.809878

**Published:** 2021-12-17

**Authors:** Lu He, Guo-Peng Wang, Jing-Ying Guo, Zhong-Rui Chen, Ke Liu, Shu-Sheng Gong

**Affiliations:** Department of Otolaryngology-Head and Neck Surgery, Beijing Friendship Hospital, Capital Medical University, Beijing, China

**Keywords:** epithelial–mesenchymal transition, vestibular, microarray, cell proliferation, hair cell, supporting cell

## Abstract

The vestibular sensory epithelium of humans and mice may degenerate into a layer of flat cells, known as flat epithelium (FE), after a severe lesion. However, the pathogenesis of vestibular FE remains unclear. To determine whether the epithelial–mesenchymal transition (EMT) participates in the formation of vestibular FE, we used a well-established mouse model in which FE was induced in the utricle by an injection of streptomycin into the inner ear. The mesenchymal and epithelial cell markers and cell proliferation were examined using immunofluorescence staining and quantitative reverse transcription polymerase chain reaction (qRT-PCR). The function of the EMT was assessed through transcriptome microarray analysis. The results demonstrated that mesenchymal cell markers (α-SMA, S100A4, vimentin, and Fn1) were upregulated in vestibular FE compared with the normal utricle. Robust cell proliferation, which was absent in the normal status, was observed in the formation of FE. Microarray analysis identified 1,227 upregulated and 962 downregulated genes in vestibular FE. Gene Ontology (GO) analysis revealed that differentially expressed genes (DEGs) were highly associated with several EMT-related GO terms, such as cell adhesion, cell migration, and extracellular matrix. Pathway enrichment analysis revealed that DEGs were enriched in the EMT-related signaling pathways, including extracellular matrix (ECM)-receptor interaction, focal adhesion, PI3K/Akt signaling pathway and cell adhesion molecule. Protein–protein interaction networks screened 20 hub genes, which were *Akt, Casp3, Col1a1, Col1a2, Fn1, Hgf, Igf1,Il1b, Irs1, Itga2, Itga5, Jun, Mapk1, Myc, Nras, Pdgfrb, Tgfb1, Thbs1*, *Trp53*, and *Col2a1*. Most of these genes are reportedly involved in the EMT process in various tissues. The mRNA expression level of hub genes was validated using qRT-PCR. In conclusion, the present study indicates that EMT plays a significant role in the formation of vestibular FE and provides an overview of transcriptome characteristics in vestibular FE.

## Introduction

Vestibular end organs, including the utricle, saccule, and cristae ampullae, are responsible for the perception of linear acceleration and head rotation. Sensory epithelia of vestibular end-organs consist of two kinds of highly differentiated cells: hair cells (HCs) and supporting cells (SCs). HCs and SCs are alternatively arranged in a special mosaic structure required for normal vestibular function. Various insults to the vestibular sensory epithelium could lead to vestibular dysfunction (McCall et al., 2009; Wang et al., 2015; Brosel et al., 2016; Isgrig et al., 2017; You et al., 2018; Zhang et al., 2020; Fu et al., 2021). Severe lesions damage both vestibular HCs and SCs and induce the sensory epithelium to be replaced by a layer of flat cells, referred to as flat epithelium (FE) (Wang et al., 2017). FE has been found in the inner ear of patients with severe deafness and/or vestibular dysfunction (Nadol and Eddington, 2006; Teufert et al., 2006; McCall et al., 2009), suggesting that FE is an important pathological change in patients with inner ear diseases. However, the pathogenesis of vestibular FE remains unknown, and there is no biological intervention for patients with FE in the inner ear. Elucidation of the molecular mechanism underlying FE formation is significant for designing therapeutic strategies for vestibular dysfunction.

The epithelial–mesenchymal transition (EMT) is a biological process (BP) that allows epithelial cells to acquire a mesenchymal cell phenotype, including migratory capacity, invasiveness, resistance to apoptosis, and increased production of extracellular matrix (ECM) components (Kalluri and Weinberg, 2009). The EMT is integral in development and wound healing, and contributes pathologically to fibrosis and cancer progression (Lamouille et al., 2014). In addition, the EMT participates in inner ear development and damage repair (Simonneau et al., 2003; Kobayashi et al., 2008; Johnen et al., 2012; Wu and Kelley, 2012). The EMT is involved in the formation of cochlear FE, which is characterized by a robust proliferative response, upregulation of mesenchymal cell markers, and cell migration (Kim and Raphael, 2007; Ladrech et al., 2017). Because the two components of the inner ear, cochlea and vestibular end-organs, share common embryonic origins and biological features, we hypothesize that the EMT also participates in the process of vestibular FE formation.

The EMT is characterized by a change in cell phenotype from epithelial to mesenchymal cells with upregulation of mesenchymal cell markers (vimentin, α-SMA, S100A4, fibronectin, N-cadherin, etc.) and downregulation of epithelial cell markers (E-cadherin, cytokeratin, and ZO-1, etc.). Thus, these factors are usually used as biomarkers to define the involvement of EMT (Kalluri and Weinberg, 2009). Recently, high-throughput screening, such as microarray and RNA-seq technologies, has enabled researchers to identify gene expression profiles in various diseases, rendering exploration of the underlying molecular mechanisms less difficult. The role of the EMT in diseases and the specific genes or signaling pathways involved have been explored using these techniques in the past decades (Puram et al., 2018). However, whether EMT participates in the inner flattening process of vestibular sensory epithelium has not been identified.

To determine the role of the EMT in the formation of vestibular FE, a high dose of streptomycin was inoculated into the mouse inner ear to induce FE in the utricle (Wang et al., 2017). Mesenchymal and epithelial cell markers and cell proliferation were assessed in normal utricle and vestibular FE using immunofluorescence staining. Then, the mRNA expression profile was examined using microarray analysis. Bioinformatics analysis was used to further analyze the biological functions of differentially expressed genes (DEGs). Finally, the representative DEGs were validated using quantitative reverse transcription polymerase chain reaction (qRT-PCR). In the present study, the role of EMT in vestibular FE formation was investigated, and the potential mechanisms underlying this process were explored.

## Results

### Expression of Mesenchymal and Epithelial Cell Markers in Utricular Flat Epithelium

**FIGURE 1:**
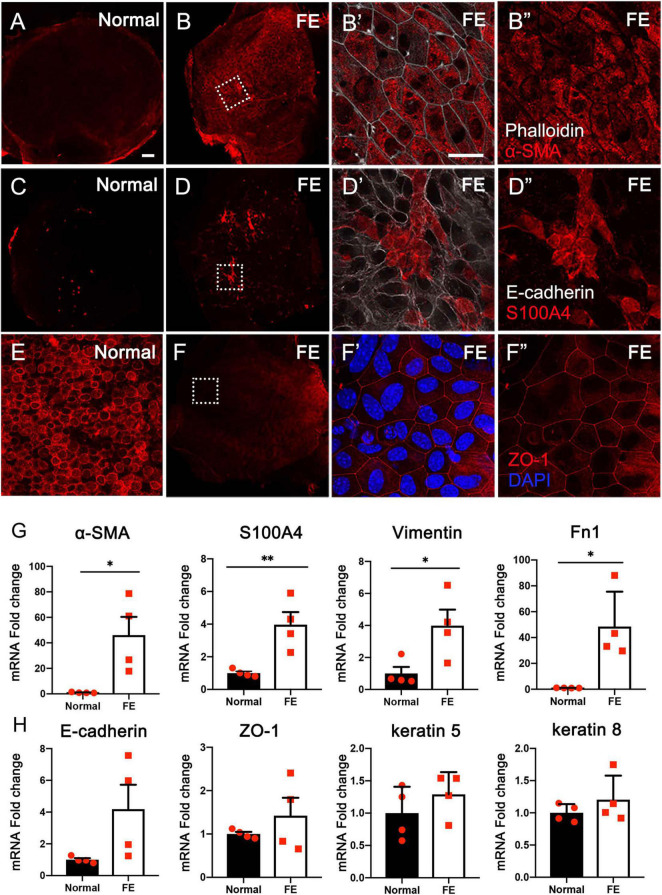
Expression of epithelial and mesenchymal cell markers in the normal utricle and flat epithelium (FE). Immunofluorescence staining of α-SMA **(A–B″)**, S100A4 **(C–D″)**, and ZO-1 **(E–F″)** showing the expression of α-SMA and S100A4 is upregulated and ZO-1 expression is downregulated in FE. High magnification images of square areas in **(B,D,F)** are shown in **(B′–B″,D′–D″,F′–F″)**, respectively. Scale bars: **(A)** (applies to **B–D**,**F**), 50 μm; **(B′)** (applies to **B″,D′,D″,E,F′, F″**), 20 μm. **(G)** qRT-PCR results revealing that the mRNA expression levels of mesenchymal cell markers (α*-SMA*, *S100A4*, *vimentin*, and *Fn1*) are significantly increased in FE compared with the normal utricle. **(H)** mRNA expression levels of epithelial cell markers (*E-cadherin*, *ZO-1*, *keratin 5*, and *keratin 8*) are not significantly different between FE and normal utricle. *P < 0.05 and ^**^P < 0.01 according to Student’s *t*-test.

To determine the potential mechanisms underlying FE formation after the loss of nearly all original epithelial cells, the expression of mesenchymal and epithelial cell markers was examined using immunofluorescence staining and qRT-PCR in the normal utricle and utricular FE samples. As shown in Figures 1A–D″, mesenchymal cell markers α-SMA and S100A4 were poorly expressed in normal utricle but highly expressed in FE. In contrast, epithelial cell marker ZO-1 was significantly expressed in the normal samples but weakly expressed in FE (Figures 1E–F″). Furthermore, the mRNA expression levels of mesenchymal cell markers, *S100A4*, α*-SMA*, *vimentin*, and *fibronectin 1* (*Fn1*) were significantly higher in FE than in the normal utricle (Figure 1G). The expression of epithelial cell markers (*E-cadherin, ZO-1, keratin 5*, and *keratin 8*) was not significantly different between the normal utricle and FE (Figure 1H).

### Robust Mitosis in Adult Mouse Utricle After Severe Damage

**FIGURE 2:**
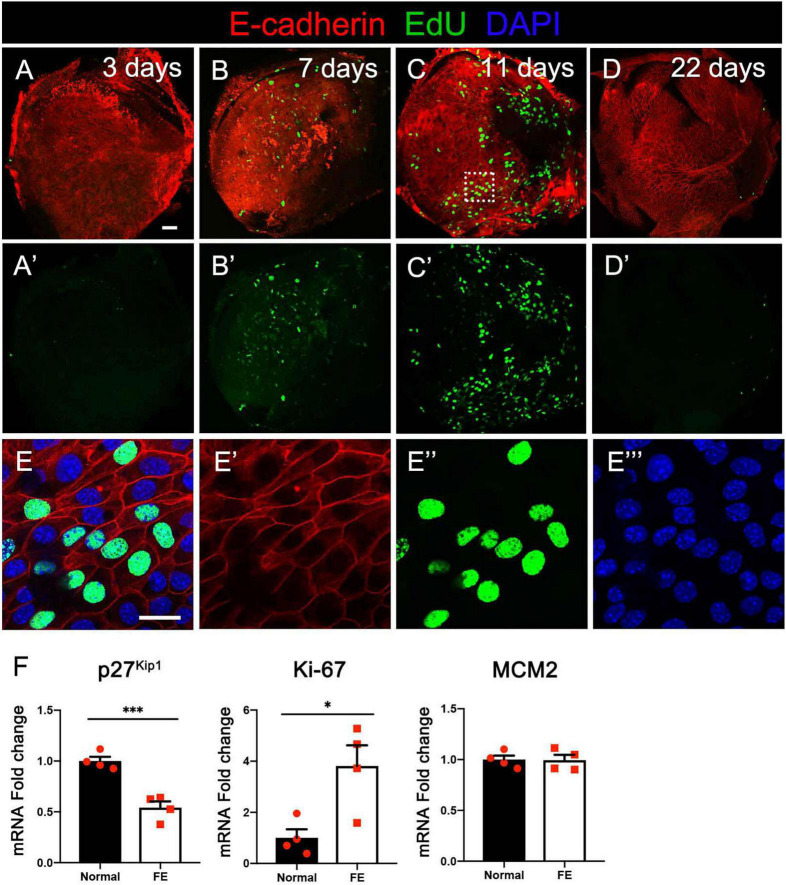
Cell division at different timepoints after severe damage to the utricular sensory epithelium. **(A–A′)** At 3 days after the lesion, the actin cytoskeleton disappeared in most areas of the epithelial layer, with a few cells labeled by EdU. **(B–B′)** The number of EdU-positive cells increased at 7 days. **(C–C′)** Robust proliferation of EdU-positive cells was detected in flat epithelium (FE) at 11 days. **(D–D′)** EdU-positive cells were not observed in the epithelial layer at 22 days. **(E–E″′)** High-magnification view of the square area in **(C)** showing EdU labeling of the nuclei of FE cells. Scale bars: **(A)** (applies to **A′–D′**), 50 μm; **(E)** (applies to **E′–E″′**), 20 μm. **(F)** qRT-PCR results revealing that the mRNA expression levels of the cell proliferation marker Ki-67 were significantly increased in FE compared with the normal utricle, and the p27^kip1^ expression level was decreased at 14 days after damage. *P < 0.05 and ^***^P < 0.001 according to Student’s *t*-test.

To evaluate if the utricular sensory epithelium possesses proliferation capacity during FE formation, the normal utricle and FE were stained with EdU to observe mitosis in the cells and with the epithelial cell marker E-cadherin to label the actin cytoskeleton. At 3 days after streptomycin injection, a few EdU-positive cells were observed (Figures 2A–A′). At 7 days after the lesion, the number of EdU-positive cells was increased (Figures 2B–B′). At 11 days after the lesion, most of the original sensory epithelium areas expressed E-cadherin, and EdU-positive cells were extensively distributed throughout the FE, indicating robust cell proliferation in the utricular FE during the early period of FE formation (Figures 2C–C′,E–E″′). At 22 days after the lesion, the epithelial cytoskeleton was completely formed, and the number of EdU-positive cells was dramatically decreased in the epithelial layer (Figures 2D–D′).

The expression levels of proliferation markers Ki-67 and MCM2 (Chow et al., 2016; Yousef et al., 2017), as well as the cell cycle inhibitor p27^kip1^ (Kim and Raphael, 2007), were evaluated and compared between the normal utricle and the utricular FE at 14 days after streptomycin injection. As shown in Figure 2F, the mRNA level of Ki-67 was significantly increased and that of p27^kip1^ decreased in FE compared with the control groups (Figure 2F).

### Microarray Analysis

**FIGURE 3:**
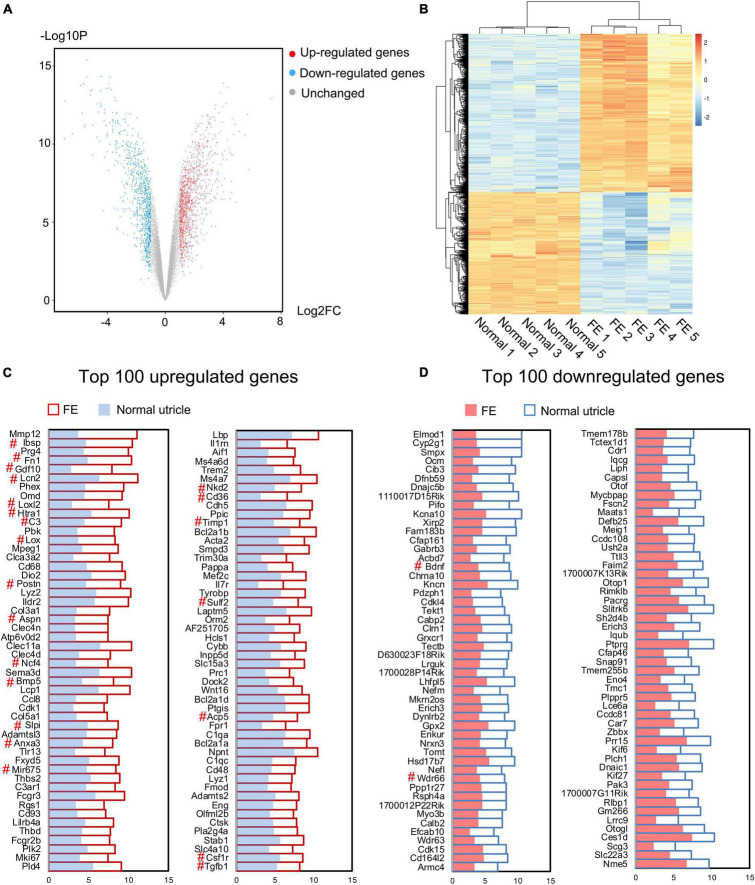
Microarray analysis of the normal utricle and flat epithelium (FE). **(A)** Volcano plot representing the whole transcriptome changes in FE compared with the normal utricle. **(B)** Hierarchical clustering showing the differentially expressed genes (DEGs). Each group has five replicates. Yellow represents the upregulated genes and blue represents the downregulated genes. **(C)** Top 100 upregulated genes in FE compared with the normal utricle. **(D)** Top 100 downregulated genes in FE compared with the normal utricle. The horizontal axis represents the expression value. The # symbol indicates genes associated with epithelial–mesenchymal transition (EMT).

To further determine the characteristics of FE transcriptomes and how the EMT is involved in the repair process of utricular sensory epithelium after severe damage, microarray analysis was performed using the Affymetrix mouse Clariom S array to analyze the transcriptomic differences between the normal utricle and FE. A total of 22,206 genes were extracted from each sample. When comparing transcripts between the normal utricle and FE, 2,189 transcripts differentially expressed (fold change > 2, *P* < 0.05) in FE were identified. Figures 3A,B show a volcano plot and hierarchical cluster analysis of the DEGs between the two groups; 1,227 upregulated and 962 downregulated genes were detected in FE samples, and heatmap analysis showed distinct differences in the mRNA expression profiles of the normal utricle and FE.

To characterize the genes most significantly differentially expressed between FE and normal utricle, the top 100 upregulated and downregulated genes were selected and are listed in Figures 3C,D. Among the DEGs, those previously reported to be associated with EMT were labeled with the # symbol; *Ibsp*, *Fn1*, *Gdf10*, *Lcn2*, *Loxl2*, *Htra1*, *C3*, *Lox*, *Postn*, *Aspn*, *Ncf4*, *Bmp5*, *Slpi*, *Anxa3*, *Mir675*, *Nkd2*, *Cd36*, *Timp1*, *Sulf2*, *Acp5*, *Csf1r*, and *Tgfb1* were upregulated in FE, whereas *Bdnf* and *Wdr66* were downregulated in FE.

### Gene Ontology Analysis

**FIGURE 4:**
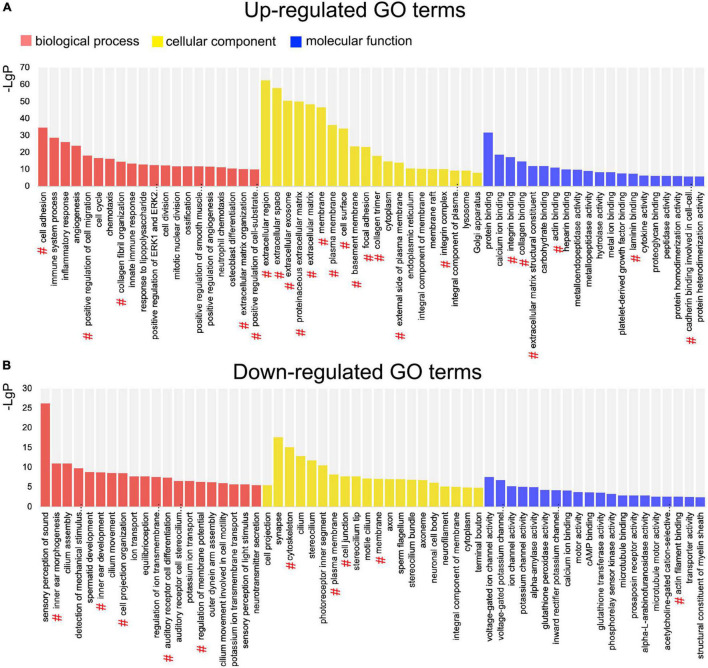
Gene Ontology (GO) enrichment analysis of differentially expressed genes (DEGs) between the normal utricle and flat epithelium (FE). **(A)** Top 20 upregulated GO terms associated with the biological process (BP; red), cellular component (CC; yellow), and molecular function (MF; blue). **(B)** Top 20 downregulated GO terms. The # symbol indicates GO terms associated with the epithelial–mesenchymal transition (EMT).

Gene Ontology (GO) enrichment analysis was performed based on the DEGs. Among the upregulated genes, 616 significant BP, 121 cellular component (CC), and 153 molecular function (MF) GO categories were detected (*P* < 0.01; Supplementary Table 1). According to the BP category results, the DEGs were significantly associated with cell adhesion and migration. In the CC category, DEGs were mainly associated with extracellular components. Among the downregulated genes, 129 significant BP, 59 CC, and 36 MF categories were detected (*P* < 0.01; Supplementary Table 2). In the BP category, DEGs were mostly associated with inner ear development and function. In the CC category, DEGs were associated with membrane, cilium, and synapse. The top 20 upregulated and the top 20 downregulated GO terms are shown in Figure 4. Among these GO terms, 34 were associated with EMT.

### Pathway Enrichment Analysis and Pathway Interaction Network Analysis

**FIGURE 5:**
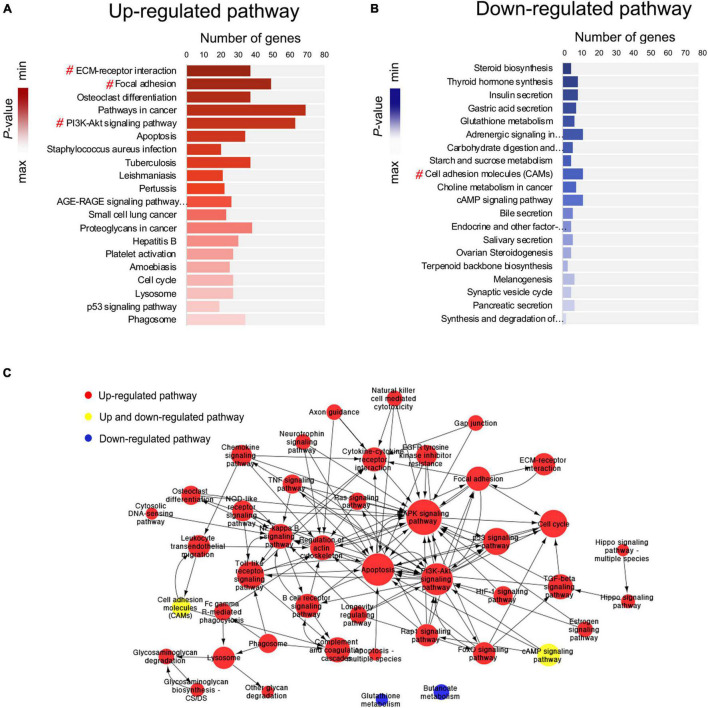
Pathway enrichment analysis and pathway interaction network analysis. **(A,B)** Pathway enrichment analysis showing the top 20 upregulated and top 20 downregulated signaling pathways. The # symbol indicates pathways associated with the epithelial–mesenchymal transition (EMT). **(C)** Pathway interaction network analysis. Nodes represent pathways, and the arrows represent an interaction target between pathways.

Pathway enrichment analysis was performed based on the KEGG (Kyoto Encyclopedia of Genes and Genomes) database. Based on the upregulated and downregulated genes, 98 and 34 signaling pathways were detected, respectively (*P* < 0.05; Supplementary Tables 3, 4). Among the top 40 significantly enriched signaling pathways (Figures 5A,B), 4 were associated with the EMT, including ECM–receptor interaction (mmu04512) (Gonzalez and Medici, 2014), focal adhesion (mmu04510) (Ji et al., 2019), PI3K/Akt signaling pathway (mmu04151) (Xu et al., 2015), and cell adhesion molecules (mmu04514) (Keller et al., 2019).

Next, pathway interaction network analysis was performed to generate an interaction network encompassing 44 significantly altered pathways; each pathway in the network was measured by counting the upstream and downstream pathways (Supplementary Table 5). A group of EMT-related signaling pathways was found to be closely associated with other pathways, including the MAPK signaling pathway (degree = 54), PI3K/Akt signaling pathway (degree = 41), TGF-β signaling pathway (degree = 17), NF-κB signaling pathway (degree = 16), regulation of actin cytoskeleton (degree = 16), and focal adhesion (degree = 16) (Figure 5C).

### Construction of the Protein–Protein Interaction Network and Screening of Hub Genes

**FIGURE 6:**
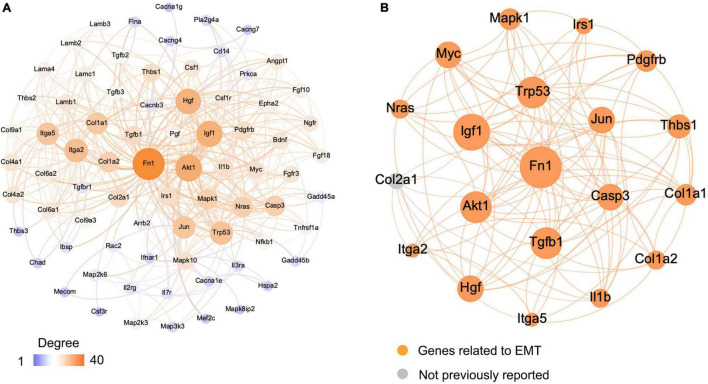
Visualization of the protein–protein interaction (PPI) network and the candidate genes. **(A)** Entire PPI network. The edges indicate the PPIs in the Search Tool for the Retrieval of Interacting Genes (STRING) database. **(B)** Identification of the candidate genes from the entire PPI network using the maximum neighborhood component (MNC) algorithm. Edges represent the protein–protein associations. The orange nodes represent the genes that have been reported to be associated with the epithelial–mesenchymal transition (EMT), and gray nodes represent genes that have not been reported previously.

The Search Tool for the Retrieval of Interacting Genes (STRING) database was used to construct a protein–protein interaction (PPI) network of selected genes. Genes involved in EMT-related signaling pathways (Figure 5) were selected to build a network using Cytoscape (v3.7.2). All the nodes and edges were mapped in the PPI network, as shown in Figure 6A. To screen hub genes from the entire PPI network, the Cytoscape plugin cytoHubba was used. A total of 20 hub genes were screened using the maximum neighborhood component (MNC) algorithm: *Akt, Casp3, Col1a1, Col1a2, Fn1, Hgf, Igf1, Il1b, Irs1, Itga2, Itga5, Jun, Mapk1, Myc, Nras, Pdgfrb, Tgfb1, Thbs1*, *Trp53*, and *Col2a1* (Figure 6B). Among those genes, 19 have been shown to participate in the EMT process in other tissues; however, an association of *Col2a1* with EMT has not been reported (Table 1).

**TABLE 1:** mRNAs involved in epithelial–mesenchymal transition (EMT) and their associated diseases or potential mechanisms.

Gene symbol	Description (disease/mechanisms)	References
*Akt1*	Breast cancer	Li et al., 2016
*Casp3*	Colon cancer cells	Zhou et al., 2018
*Col1a1*	Colorectal cancer	Zhang et al., 2018
*Col1a2*	Colon cancer	Zhu et al., 2020
*Fn1*	ECM glycoprotein; enhances cell invasion and migration	Ritzenthaler et al., 2008; Sen et al., 2010
*Hgf*	Activates downstream pathways including MAPK and PI3K	Liu et al., 2017
*Igf1*	Activator of EMT in several types of cancer through signaling pathways including JNK, MAPK, and PI3K/Akt	Haisa, 2013
*Il1b*	Epithelial and cancer cells	Wang et al., 2018
*Irs1*	Regulates the expression of E-cadherin; promotes Wnt-mediated EMT	Geng et al., 2014
*Itga2*	Prostate cancer	Gaballa et al., 2020
*Itga5*	Oral squamous carcinoma	Deng et al., 2019
*Jun*	Human nasopharyngeal carcinoma cells	Lin et al., 2018
*Mapk1*	Inhibits invasion and metastasis	Li et al., 2015
*Myc*	Breast cancer	Yin et al., 2017
*Nras*	Drives a switch in EMT transcriptional factor expression	Caramel et al., 2013
*Pdgfrb*	Tongue squamous carcinoma	Zhang et al., 2016
*Tgfb1*	Regulates genes associated with ECM, cellular motility, and tight junctions	Zarzynska, 2014
*Thbs1*	Major activator of TGF-β	Murphy-Ullrich and Poczatek, 2000
*Trp53*	Regulates specific miRNAs	Chang et al., 2011
*Col2a1*	Not previously reported	

### Quantitative Reverse Transcription Polymerase Chain Reaction Validation

**FIGURE 7:**
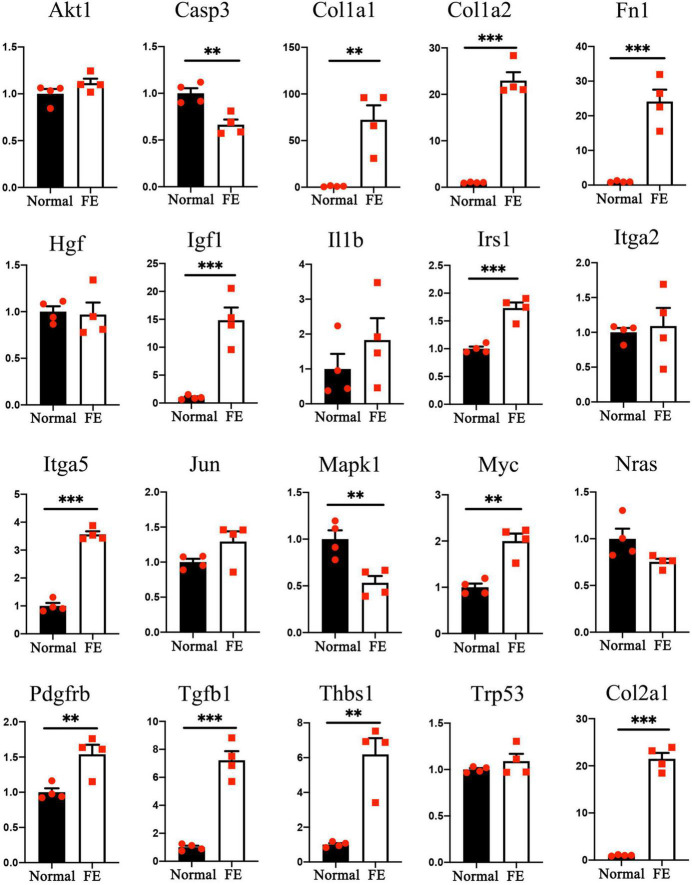
Quantitative reverse transcription polymerase chain reaction analysis of the 20 hub genes. ^**^P < 0.01 and ^***^P < 0.001 according to Student’s *t*-test.

The gene expression levels of the 20 hub genes were examined between FE and normal utricle using qRT-PCR. Compared with normal utricle, mRNA expression levels of *Casp3, Col1a1, Col1a2, Col2a1, Fn1, Igf1, Irs1, Itga5, Mapk1, Myc, Pdgfrb, Tgfb1*, and *Thbs1* were significantly upregulated in FE (Figure 7). The expression levels of the rest genes showed no significant differences in FE compared with normal utricle.

## Discussion

The present study results revealed that mesenchymal cell markers (α-SMA, S100A4, vimentin, and Fn1) were upregulated, and robust cell proliferation was detected, during the formation of vestibular FE. Furthermore, microarray analysis further confirmed that multiple EMT-related pathways and genes were involved in this process. These findings demonstrated that EMT participated in the epithelial reorganization of vestibular sensory epithelium after severe damage induced by aminoglycoside antibiotics.

Epithelial–mesenchymal transition is a physiological process that enables epithelial cells to acquire a mesenchymal cell phenotype (Kalluri and Weinberg, 2009). The expression changes in specific markers are used to confirm EMT (Nieto et al., 2016). Vimentin is a type of intermediate filament and a commonly used marker of mesenchymal properties (Thiery et al., 2009). Vimentin is upregulated in several wound healing models (Cheng and Eriksson, 2017). In the inner ear, vimentin is expressed in the SCs of normal cochlea and might contribute to the process of scar formation after HC loss (Oesterle et al., 1990; Ladrech et al., 2017). Vimentin is also present in the cochlear FE (Ladrech et al., 2017). In the present study, vimentin expression was significantly upregulated in the vestibular FE compared with the normal utricle (Figure 1G). In addition, α-SMA, S100A4, and Fn1 are mesenchymal markers commonly used to evaluate EMT (Kalluri and Weinberg, 2009). In the present study, α-SMA and S100A4 were poorly expressed in the normal utricle, while they showed evident expression in FE (Figures 1A–D″). The mRNA expression levels of α*-SMA*, *S100A4*, and *Fn1* were significantly increased in FE compared with normal utricle (Figure 1G), indicating that expression of mesenchymal cell markers is increased in the vestibular FE.

E-cadherin and cytokeratin are two commonly used markers of epithelial properties (Nieto et al., 2016). In the present study, immunostaining results showed that the epithelial markers E-cadherin and ZO-1 were expressed in the vestibular FE (Figures 1C–F″). Furthermore, qRT-PCR revealed no significant difference in the expression of epithelial cell markers (E-cadherin, keratin 5, keratin 8, and ZO-1) between FE and normal utricle (Figure 1H). In the cochlea, E-cadherin was strongly expressed in both normal sensory epithelium and FE, although a drastic downregulation was found after aminoglycoside ototoxicity (Ladrech et al., 2017). These data indicate that the inner ear FE possesses both epithelial and mesenchymal phenotypes. The hybrid phenotype, which is involved in various pathophysiological processes and diseases, is considered to be resulted from partial EMT (Hahn et al., 2016; Nieto et al., 2016; Ladrech et al., 2017; Takahashi et al., 2019). The hybrid phenotype of FE cells may facilitate maintenance of the epithelial barrier in the inner ear.

In the present study, robust cell proliferation was found during the early stage of vestibular FE after the damage (Figure 2), which was similar to cell proliferation in the formation of cochlear FE (Kim and Raphael, 2007). EMT has been reported to induce stem cell properties, including proliferation and self-renewal in various types of tissues (Jessen and Arthur-Farraj, 2019; Wang and Unternaehrer, 2019). During the cutaneous wound healing process, partial EMT induces epithelial cells undergoing proliferation and migration (Haensel and Dai, 2018). Complex mechanisms may underlie the activity of EMT and proliferation. The PI3K/Akt pathway and *Myc* gene are involved in this process (King et al., 2015; Yu and Cui, 2016). PI3K/Akt signaling plays a key role in the regulation of cell proliferation. Akt is the major downstream target of PI3K. Akt overexpression decreases the level of mitosis marker p27^kip1^ and results in enhanced proliferation (Shen et al., 2020). In the present study, *Akt1* upregulation (Figure 6B) and *p27^kip1^* downregulation (Figure 2F) were detected at 14 days after the damage of the vestibular sensory epithelium. In addition, *Myc* was significantly upregulated and identified as one of the 20 hub genes (Figure 7 and Table 1). A major role of *Myc* is control of cell proliferation (Bretones et al., 2015), and *Myc* could lead to the proliferation of mature SCs in adult mice (Shu et al., 2019). The role of such key genes in cell proliferation in vestibular FE needs further investigation.

A multi-step integrative bioinformatics analysis was performed to explore the EMT function in vestibular FE. Based on GO analysis, DEGs were significantly enriched in GO terms associated with cell adhesion, cell migration, and extracellular components. Changes in cell adhesion molecules and acquisition of migratory ability are major characteristics of the EMT process (Nieto et al., 2016). Based on KEGG pathway enrichment analysis and pathway interaction network analysis, the PI3K/Akt signaling pathway was among the core positions in the pathway interaction network, indicating that this pathway plays an important role in the formation of vestibular FE. PI3K/Akt pathway, an important signaling pathway involved in the EMT process, may directly induce EMT by regulating transcription factors or other signaling pathways (Xu et al., 2015). PI3K/Akt signaling accelerates EMT and wound healing in epithelial tissue (Xiao et al., 2017).

In the present study, 19 of 20 hub genes selected using the MNC algorithm have been reportedly involved in EMT in various tissues (Figure 6B and Table 1). Among them, the expression of 13 hub genes changed significantly by qRT-PCR (Figure 7). Some of them are involved in the proliferation, development, nerve regeneration and protection of the inner ear. *Fn1* promotes cell invasion and migration by regulating cell adhesion and ECM proteins (Ritzenthaler et al., 2008; Sen et al., 2010). Fn1 may be involved in EMT process during cochlear fibrosis (Jia et al., 2016). The present study revealed that *Fn1* was in the core position in the PPI network (Figure 6B), indicating that *Fn1* might be a key regulator of the EMT process in vestibular FE. *Igf1* is an activator of EMT through several signaling pathways (Haisa, 2013). In the developing inner ear, *Igf1* is highly expressed during otic development, and it could protect HCs from ototoxic damage and increase the HC proliferation rate (Varela-Nieto et al., 2004). *Myc* and *Casp3* regulate cell proliferation and apoptosis in the inner ear respectively (Van De Water et al., 2004; Hu et al., 2021). *Thbs1* promotes EMT through activation of TGF-β and plays an important role in the development of cochlear afferent synapse (Jayachandran et al., 2014; Mendus et al., 2014). Altogether, these studies suggest that the hub genes relevant to the inner ear, such as *Fn1, Igf1, Myc, Casp3*, and *Thbs1*, may play a significant role during EMT process of vestibular FE. However, the exact pathophysiological mechanisms need to be further explored.

In conclusion, the present study results showed that upregulation of mesenchymal cell markers, downregulation of epithelial cell markers, and robust cell proliferation were detected in vestibular FE. Furthermore, this is the first study in which the transcriptome profile of vestibular FE was reported. Microarray analysis showed a significant difference in the transcriptome profiles between the normal utricle and FE, with many genes associated with EMT. In addition, a group of GO terms and pathways were associated with EMT. Altogether, these findings demonstrated that the EMT plays a significant role in the transition from normal vestibular sensory epithelium to FE induced by aminoglycoside antibiotics. Additional research is needed to determine the probable biological intervention strategies of FE based on the transcriptome features identified in the present study.

## Materials and Methods

### Animals and Surgery

FVB/N mice (4–5-week-old) were purchased from SPF Biotechnology (Beijing, China) and housed in the Laboratory Animal Department of Capital Medical University. All animal experiments were approved by the Animal Care and Use Committee of Capital Medical University of China.

When mice were 6 weeks old, 400 μg of streptomycin (Sigma, St. Louis, MO, United States) dissolved in normal saline (400 g/L, 1 μL), was inoculated into the inner ear through the posterior semicircular canal to induce a severe lesion in the mouse utricle. The surgery was performed as described previously (Guo et al., 2018).

### Immunofluorescence Staining

Mice were euthanized 2 weeks after the surgery. The temporal bones were fixed in 4% paraformaldehyde in phosphate-buffered saline (PBS) for 2 h. The utricles were dissected out and treated with 0.3% Triton X-100 (Sigma) and 5% normal goat serum (ZSGB-BIO, Beijing, China) in PBS for 2 h at room temperature. Samples were incubated with primary antibody at 4°C overnight. We used the following primary antibodies: mouse anti-α-SMA (diluted 1:300, Sigma), rabbit anti-S100A4 (diluted 1:300, Sigma), E-cadherin (diluted 1:200, BD Biosciences, San Jose, CA, United States), and ZO-1 (diluted 1:200, Invitrogen, Carlsbad, CA, United States). After rinsing in PBS for three times, samples were incubated in fluorescence-labeled secondary antibodies tagged with Alexa Fluor 568 or 647 (diluted 1:300, Invitrogen) for 2 h at room temperature. Alexa Fluor 647-conjugated phalloidin (diluted 1:300, Invitrogen) was used for F-actin labeling. Samples were incubated in the DNA-binding fluorescent stain 4′,6-diamidino-2-phenylindole (diluted 1:1000, AppliChem, Darmstadt, Germany) for 5 min to label nuclei.

To detect cells entering the cell cycle at different time points after streptomycin administration, EdU (20 mg/kg body weight, Invitrogen) was given once intraperitoneally at 3, 7, 11, or 22 days after surgery. Mice were euthanized 24 h after EdU administration. The Click-iT EdU Cell Proliferation Kit (Invitrogen) was used to perform the click reaction. Samples were then treated with primary and secondary antibodies as described above.

Samples were mounted on glass slides with Fluoromount-G (Southern Biotech, Birmingham, AL, United States) and examined with a scanning confocal microscope (Leica Camera AG, Solms, Hessen, Germany). Images were labeled and spaced using Photoshop (Adobe Systems, San Jose, CA, United States).

### mRNA Microarray Analysis

The utricle tissues were collected for microarray analysis 2 weeks after surgery. Total RNA was isolated using the Qiagen RNeasy Mini Kit (Qiagen, Hilden, Germany). Each group contained five samples, and each sample had three utricles. Microarray analysis was performed by CapitalBio (Beijing, China). The Affymetrix mouse Clariom S Array (Affymetrix, Santa Clara, CA, United States) was used for hybridization. Student’s *t*-test was applied for comparison of the two groups. Genes with a fold change > 2 and a *P*-value < 0.05 were considered significantly different. The dataset was submitted to Gene Expression Omnibus (GSE179063).

### Bioinformatics Analysis

Gene Ontology analysis was performed for the DEGs. The distribution of genes in the three ontologies, including BP, CC, and MF, reflects the effects of the particular genes. GO statistical analysis was performed using Fisher’s exact test. A *P*-value < 0.01 was used as cutoff to select significantly enriched GO terms.

Pathway analysis was used to find significantly enriched functional pathways according to the KEGG database. Fisher’s exact test was used to identify the enriched pathways, and *P*-value < 0.05 was used as cutoff to select significantly enriched pathways.

A pathway interaction network was constructed based on the KEGG analysis to determine the relationships between enriched pathways. The degree represents the relationship between one pathway and the pathways around it. Cytoscape (v3.7.2) (Shannon et al., 2003) was used to draw the network diagram.

To better illustrate the interactions of the DEGs, the STRING^1^ database was used to predict the associations of the selected genes. The parameter was set as interaction score ≥0.5. The PPI network was constructed and visualized using Cytoscape (v3.7.2). The key DEGs were selected using the MNC. The MNC of each node was calculated using cytoHubba, a Cytoscape plugin, and the genes with the top 20 MNC values were considered hub genes.

### Quantitative Reverse Transcription Polymerase Chain Reaction Analysis

Four independent RNA pools were prepared for each group, and three utricles were dissected in RNAlater (Qiagen, Hilden, Germany). TRIzol reagent (Life Technologies, Carlsbad, CA, United States) was used to isolate total RNA. cDNA was synthesized using FastQuant RT Super Mix reverse transcription (Tiangen Biotech Co., Ltd.). qRT-PCR was performed using FastStart Universal SYBR Green reagent (Bio-Rad Laboratories, Hercules, CA, United States) and primers. The mouse glyceraldehyde-3-phosphate dehydrogenase (*GAPDH*) gene was used as a reference. The 2^–ΔΔCT^ method was applied to calculate changes in mRNA expression levels of the candidate genes.

### Statistical Analysis

Statistical analysis was performed using GraphPad Prism 8 (GraphPad Software, Inc., La Jolla, CA, United States). The mRNA expression levels detected by qRT-PCR were expressed as means ± SE and analyzed using unpaired Student’s *t*-test. Differences were considered statistically significant at *P* < 0.05.

## Data Availability Statement

The datasets presented in this study can be found in online repositories. The names of the repository/repositories and accession number(s) can be found below: https://www.ncbi.nlm.nih.gov/geo/, GSE179063.

## Ethics Statement

The animal study was reviewed and approved by the Animal Care and Use Committee of Capital Medical University of China.

## Author Contributions

LH contributed to the conceptualization and methodology of the study and wrote the original draft. G-PW contributed to the conceptualization and methodology of the study, manuscript writing, reviewing, and editing. J-YG completed the formal analysis and data curation. Z-RC performed the investigation and formal analysis. KL was responsible for the software and validation. S-SG provided the conceptualization, writing, reviewing, and supervision. All authors contributed to the article and approved the submitted version.

## Conflict of Interest

The authors declare that the research was conducted in the absence of any commercial or financial relationships that could be construed as a potential conflict of interest.

## Publisher’s Note

All claims expressed in this article are solely those of the authors and do not necessarily represent those of their affiliated organizations, or those of the publisher, the editors and the reviewers. Any product that may be evaluated in this article, or claim that may be made by its manufacturer, is not guaranteed or endorsed by the publisher.

## Footnotes

1
https://string-db.org/
